# Genetic Predisposition and Mitochondrial Dysfunction in Sudden Cardiac Death: Role of MCU Complex Genetic Variations

**DOI:** 10.3390/cells14100728

**Published:** 2025-05-16

**Authors:** Haoliang Meng, Yan He, Yukun Rui, Mengqi Cai, Dongke Fu, Wanli Bi, Bin Luo, Yuzhen Gao

**Affiliations:** 1 Department of Forensic Medicine, Suzhou Medical College, Soochow University, Suzhou 215006, China; 20234221083@stu.suda.edu.cn (H.M.); r_yukun18@163.com (Y.R.); mengqicai0920@163.com (M.C.); 2 Jiangsu Key Laboratory of Preventive and Translational Medicine for Geriatric Diseases, Department of Epidemiology, School of Public Health, Suzhou Medical College, Soochow University, Suzhou 215006, China; yhe@suda.edu.cn; 3 NuHigh Biotechnologies Co., Ltd., Suzhou 215000, China; dongke.fu@nuhighbio.com (D.F.); wanli.bi@nuhighbio.com (W.B.); 4 Department of Forensic Pathology, Zhongshan School of Medicine, Sun Yat-sen University, Guangzhou 510080, China

**Keywords:** sudden cardiac death, coronary artery disease, mitochondrial calcium uniporter (MCU), indel variant, *SMDT1*

## Abstract

Sudden cardiac death (SCD) is a major cause of cardiovascular mortality, with coronary artery disease-related SCD (SCD-CAD) being the most prevalent form. Genetic factors and mitochondrial dysfunction, particularly in calcium homeostasis, are critical in SCD-CAD. However, the specific genetic factors linked to mitochondrial dysfunction in SCD-CAD remain poorly understood. In this case-control study, we analyzed 229 SCD-CAD cases and 598 controls from a Southern Han Chinese population, focusing on 12 insertion-deletion (indel) variants across six mitochondrial calcium uniporter (MCU) complex genes. We used capillary electrophoresis-based multiplex genotyping and performed logistic regression and haplotype analyses to assess the association of these variants with SCD-CAD susceptibility. Four significant indel variants and three risk-associated haplotypes were identified. Two of these indels were previously validated in the GWAS catalog as strongly linked to cardiac disorders. Additionally, Mendelian randomization (MR) analysis revealed a causal relationship between elevated levels of the *SMDT1*-encoded MCU regulator and increased risks of cardiovascular diseases, including coronary atherosclerosis, myocardial infarction, and cardiomyopathy. These findings highlight the role of MCU complex variants in SCD-CAD susceptibility and suggest their potential as biomarkers for cardiovascular risk stratification. Further research with larger cohorts is needed to confirm these results and explore underlying mechanisms.

## 1. Introduction

Sudden cardiac death (SCD) is defined as an unexpected natural death caused by cardiovascular conditions, occurring within a short timeframe (typically within one hour of symptom onset) and in the absence of prior fatal symptoms. It accounts for approximately 50% of all cardiovascular-related deaths [1,2]. Epidemiological studies estimate the overall incidence of SCD to be 41.8 per 100,000 individuals across four regions of China, with coronary artery disease (CAD)-related SCD (SCD-CAD) being the primary cause [3,4]. This condition poses a significant public health burden. SCD-CAD is a severe, multifactorial disorder with a complex etiology influenced by genetic, environmental, and acquired factors. It is worth noting that SCD exhibits age-dependent etiologies. In younger individuals, genetic factors are the predominant cause, leading to channelopathies and cardiomyopathies, which result from mutations in genes encoding ion channels or structural proteins that impair cardiac function. In contrast, adult SCD is primarily associated with ischemic heart disease, including myocardial infarction and CAD [4]. These age-related differences underscore the importance of considering age-specific pathophysiological mechanisms in understanding the varying risks of SCD across different age groups. The immediate and unexpected nature of SCD symptoms, coupled with the lack of definitive findings in approximately 40% of conventional autopsy cases (termed “negative autopsy”), highlights the urgent need for improved diagnostic methods [5]. Advances in molecular diagnostics and autopsy techniques have revealed that rare genetic variants are major contributors to SCD susceptibility and play a critical role in its prediction and prevention [6,7,8,9]. Identifying novel genetic risk factors associated with SCD-CAD is, therefore, essential for facilitating effective screening and risk stratification.

The human heart demands substantial energy to sustain each cardiac cycle, with mitochondria playing a pivotal role in ATP production to support its essential functions. In cardiomyocytes, mitochondria occupy approximately one-third of the cellular volume and generate over 95% of the required ATP [10]. Under physiological conditions, mitochondrial ATP production relies on the mitochondrial calcium uniporter (MCU) complex, located in the inner mitochondrial membrane (IMM), for Ca^2+^ transfer into the mitochondrial matrix. This pathway serves as the primary mechanism for mitochondrial Ca^2+^ uptake [11]. In mammals, the MCU complex comprises several components, including the pore-forming MCU protein, the dominant-negative β-subunit (MCUB), the mitochondrial calcium uptake (MICU) family (MICU1, MICU2, MICU3), the essential MCU regulator (EMRE), and MCU regulator 1 (MCUR1). Collectively, these elements maintain mitochondrial Ca^2+^ homeostasis [11]. Mitochondrial Ca^2+^ concentration ([Ca^2+^]_m_) within physiological levels enhances ATP production; however, deviations from this range can elevate reactive oxygen species (ROS) production, impairing mitochondrial function and potentially triggering cell death [12,13,14]. Impaired MCU function has been implicated in various cardiovascular conditions, including heart failure, atherosclerosis, arrhythmias, and ischemia-reperfusion (IR) injury [15,16,17,18]. Cardiomyopathy is a major substrate of SCD, with genetic mutations playing a key role. For example, mutations in the desmin (*DES*) gene (OMIM ID: 125660) impair filament formation, disrupt intercalated disk localization, and compromise cellular integrity, leading to electrical instability and an increased risk of SCD [19,20]. Desmin, encoded by the *DES* gene, is particularly noteworthy as it forms a structural network coupled with mitochondria. This interaction highlights its role in maintaining cellular architecture and function, emphasizing its importance in cardiomyopathy-related arrhythmias and the mitochondrial dysfunction observed in SCD. However, the role of upregulating or downregulating MCU complex activity in the progression of cardiovascular diseases remains controversial. Evidence suggests that downregulating MCU activity can prevent mitochondrial Ca^2+^ overload, thereby preventing mitochondrial dysfunction and disease progression [21,22]. Conversely, some studies indicate that upregulating MCU activity may be beneficial [18,23]. These previous studies have suggested there is some potential association between the MCU complex and cardiovascular diseases. However, determining a causal relationship through traditional observational studies is challenging due to the presence of multiple confounding factors.

Capillary electrophoresis (CE) and multiplex dye-labeling fluorescence techniques are widely utilized in forensic genetics, such as short tandem repeat (STR) typing, due to their high efficiency and cost-effectiveness [24]. Similar to STRs, insertions and deletions (indels) are length polymorphisms and represent the second most prevalent type of DNA marker after single nucleotide polymorphisms (SNPs). Indel polymorphisms have been shown to significantly influence human biology and disease susceptibility [25]. In this study, we employed a systematic variant screening strategy and developed a novel multiplex amplification system by using a CE platform to genotype 12 candidate indel variants across six core MCU complex genes in a Southern Han Chinese cohort. Logistic regression analysis was used to investigate the role of indel variants and haplotypes in influencing SCD-CAD risk. Additionally, bidirectional Mendelian randomization (MR) analyses were performed using summary statistics from the largest genome-wide association studies (GWAS) on cardiovascular diseases, coronary atherosclerosis, myocardial infarction, and cardiomyopathy. These analyses were conducted to assess the causal effect of MCU levels on the risk of these conditions.

## 2. Materials and Methods

### 2.1. Ethics Statement

The research project was approved by the Ethical Committee of Soochow University (approval number: ME81772029). Written informed consent was obtained from the relatives of each participant prior to the study. No further institutional review board approval was required for MR analysis as all summary-level GWAS data included in this study were derived from the IEU GWAS database (https://gwas.mrcieu.ac.uk/, accessed on 11 October 2024), which has received ethical approval from the relevant institutional review boards, with informed consent provided by participants.

### 2.2. Study Populations in Case-Control Study

A total of 598 healthy controls and 229 cases, all genetically unrelated ethnic Han Chinese, were recruited for this study. Blood samples for SCD-CAD were collected from the following institutions during forensic autopsy: Soochow University, Institute of Forensic Science, Ministry of Justice, and Medicolegal Expertise Center of Sun Yat-sen University. The SCD cases were validated through comprehensive forensic pathological investigations, following criteria described previously [26,27]. In summary, each case underwent thorough forensic pathological examination to confirm that coronary heart disease was the primary cause of SCD. Apart from varying levels of coronary atherosclerosis, no other fatal conditions were identified. Moreover, forensic toxicological analyses were conducted in all cases to exclude potential poisoning. Healthy controls matched by age (within ±5 years) were recruited from the same region during the same timeframe as the recruitment of cases, while individuals with any positive family history of SCD or cardiovascular diseases were excluded.

### 2.3. Selection of MCU Gene Variants

Significant single-tissue expression quantitative trait locus (eQTLs) for six core MCU complex genes, including *SMDT1* (OMIM ID: 615588), *MCU* (OMIM ID: 614197), *MICU1* (OMIM ID: 605084), *MICU2* (OMIM ID: 610632), *MICU3* (OMIM ID: 610633), *MCUB* (OMIM ID: 620702), in all tissues of the Genotype-Tissue Expression (GTEx) database (5394 SNPs and Indels) were used to select variants. In order to circumvent the potential for false positives and evolutionary disadvantage, a minor allele frequency (MAF) > 0.10 was set for variant selection. Given the discriminable ability of CE, the length difference between two alleles of indels should be >1 base pair (bp). After selection, 7% non-denaturing polyacrylamide gel electrophoresis (PAGE) and silver staining were employed to determine the characteristics and preliminary frequency of selected indel variants [28]. The validated indel variants were ranked based on 4 comprehensive databases, including 3DSNP v2.0, RegulomeDB v.2, HaploReg v4.2 and JASPAR CORE 2022. Linkage disequilibrium (LD) analysis was performed using Haploview software (version 4.2), and the genotype of variants used for LD were obtained from Ensemble Release 111 (January 2024).

### 2.4. Multiplex Genotyping

For primer design, the MFEprimer-3.1 was employed to ensure that the potential primer dimers and secondary hairpin structures were not included in mixed primers [29]. The Blood DNA Kits or Blood Spots DNA Kits (TIANGEN, Beijing, China) were used to extract genomic DNA from blood samples. The amplification was performed on a Perkin Elmer 9700 Thermal Cycler (PerkinElmer, Waltham, MA, USA). Amplified products were analyzed using the ABI 3500xL Genetic Analyzer (Applied Biosystems, Foster City, CA, USA) equipped with the NH-6V dye set (Nuhigh, Suzhou, China) and POP4 polymers. Genotyping data were obtained by analyzing the electrophoretic results with GeneMapper™ ID-X V1.6 software (Applied Biosystems). Allele peaks were defined using an analytical threshold of 150 relative fluorescence units (RFU). The specific methods for DNA extraction, PCR reactions and genotyping can be found in the Supplementary Table S1.

### 2.5. Statistical Analysis of Case-Control Study and Haplotype Analysis

After determining allele counts and frequencies through electrophoresis analysis, the Hardy–Weinberg equilibrium (HWE) and chi-square test were applied to assess the representativeness of the control group. Statistical analyses were performed using IBM SPSS Statistics 27 (SPSS Inc., Chicago, IL, USA). Binary logistic regression analysis was conducted to calculate odds ratios (ORs) and 95% confidence intervals (95% CIs) to evaluate the association between indel variants and the risk of SCD-CAD. Basic information and genotypes of the variants were documented in ped and info files, respectively, for subsequent haplotype analysis. Haplotype analyses of the candidate indel variants were conducted using Haploview v4.2 software, with chi-square tests employed for statistical evaluation. To ensure statistical significance, the Bonferroni method was applied for multiple testing on the haplotype results. All tests of the case-control study were two-sided, and *p*-values < 0.05 were considered statistically significant, and the tests of haplotype analysis *p*-values < Bonferroni correction were considered statistically significant.

### 2.6. Bioinformatic Analysis of Positive Indel Variants

After identifying indel variants associated with SCD-CAD susceptibility, the eQTL analysis was performed in the GTEx database. Subsequently, three comprehensive GWAS databases, including Finngen, UK Biobank (UKB), and the Million Veteran Program (MVP), were used to further investigate the relationships between positive indel variants and various cardiovascular-related diseases. Based on original data from GWAS, the analyses were performed using R software (version 4.4.1, https://www.datavis.ca/R/, accessed on 5 December 2024).

### 2.7. MR Analysis of SMDT1-Encoded Mitochondrial MCU Regulator Levels with Cardiovascular Diseases

The MR analysis adheres to the Strengthening the Reporting of Observational Studies in Epidemiology Using Mendelian Randomization (STROBE-MR) guidelines [30]. The data of *SMDT1*-encoded mitochondrial MCU regulator levels were obtained from a previous GWAS [31]. Data for outcomes, including cardiovascular diseases, coronary atherosclerosis, myocardial infarction and cardiomyopathy, were sourced from publicly available GWAS data from the FinnGen consortium. The detailed data sources are shown in Supplementary Table S2.

To identify relevant genetic instruments (IVs) associated with *SMDT1*-encoded mitochondrial MCU regulator levels, we initially selected SNPs reaching genome-wide significance (*p* < 5 × 10⁻^6^). To minimize potential bias due to LD, we applied a clumping process with a strict LD threshold (r^2^ < 0.001) within a 10,000 kilobase window. The strength of the IVs were assessed using the F-statistic, with values > 10 indicating a low likelihood of weak instrument bias [32]. Next, we harmonized the exposure and outcome datasets to ensure consistent effect alleles and removed palindromic SNPs with intermediate allele frequencies. After this stringent selection process, 13 SNPs significantly associated with *SMDT1*-encoded mitochondrial MCU regulator levels were retained for analysis.

Following SNP selection, we employed the inverse-variance weighted (IVW) method as our primary approach to examine genetic associations between *SMDT1*-encoded mitochondrial MCU regulator levels and multiple cardiovascular phenotypes. The IVW method combines Wald ratio estimates through meta-analysis, offering robust causal inference while remaining vulnerable to horizontal pleiotropy and outlier effects [33]. To evaluate potential biases, we conducted Cochran’s Q test for heterogeneity assessment [34], MR-Egger intercept test and MR-PRESSO global test for horizontal pleiotropy detection [35,36]. Depending on these results, we applied either random-effects or fixed-effects IVW models accordingly [37]. All analyses were performed using R (version 4.4.1) with the “TwoSampleMR” and “MRPRESSO” packages, while statistical power was estimated via the mRnd online platform [38]. A statistically significant association (*p* < 0.05) in the IVW analysis was considered evidence of potential causality.

## 3. Results

### 3.1. Selection of Candidate Indel Variants

**Figure 1:**
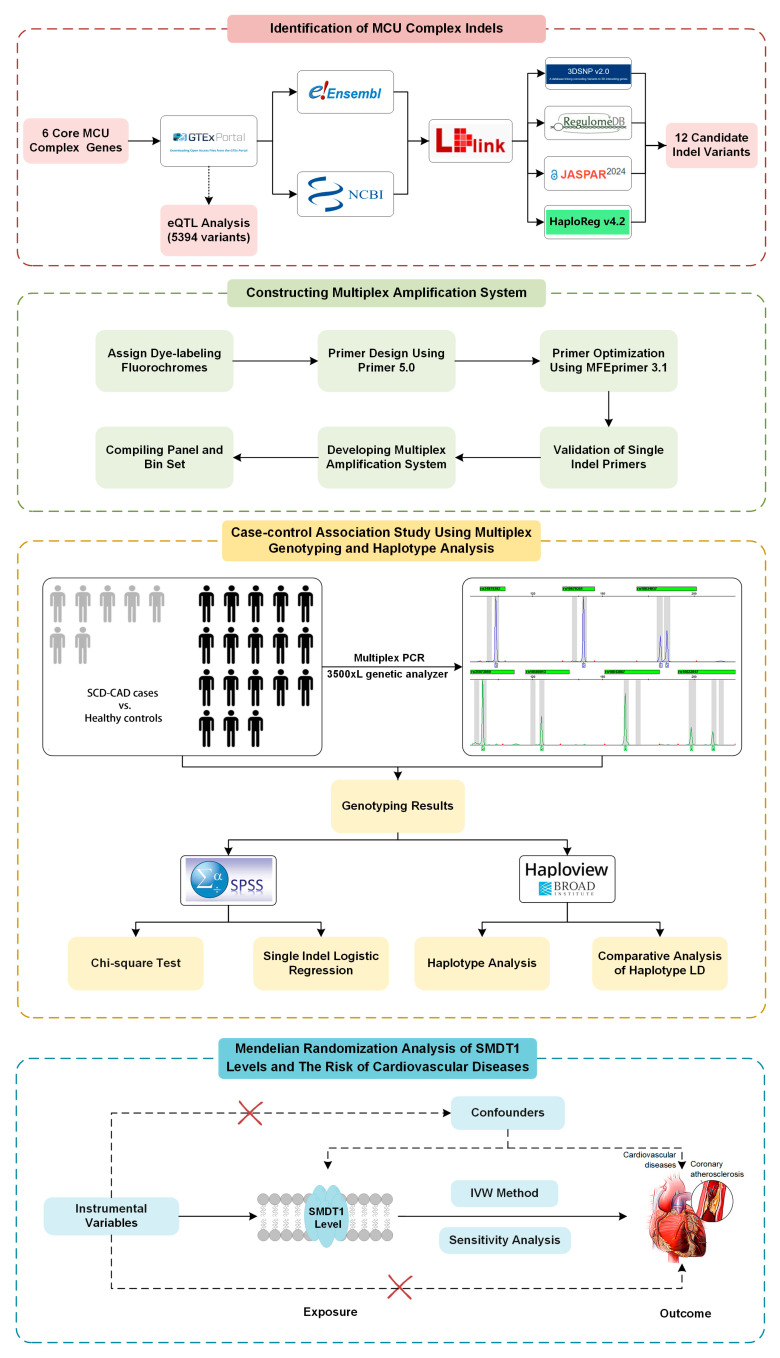
Study design of MCU complex variants and their associations with SCD-CAD.

The study design is illustrated in Figure 1. Using the GTEx database, we acquired 5394 variants with significant single-tissue eQTLs for six core genes (*MCU*, *MICU1*, *MICU2*, *MICU3*, *SMDT1*, *MCUB*) from the MCU complex. Following screening and LD analysis, we identified 39 indel variants with an indel length > 1 bp and MAF > 0.10. Subsequently, we selected 12 representative indel variants associated with MCU complex gene expression based on PAGE analysis and ranking scores from four databases. These included rs34979382, rs139522, rs140490511, rs59575728 in *SMDT1*, rs10670351 and rs10634037 in *MICU1*, rs35073080, rs10580013, rs10622847 in *MICU2*, rs10680396 in *MICU3*, rs10643067, rs5860983 in *MCUB*. Detailed information and ranking scores for these 12 validated indel variants are provided in Table 1 and Supplementary Table S3, respectively.

### 3.2. Construction and Optimization of Multiplex Amplification System

**Figure 2:**
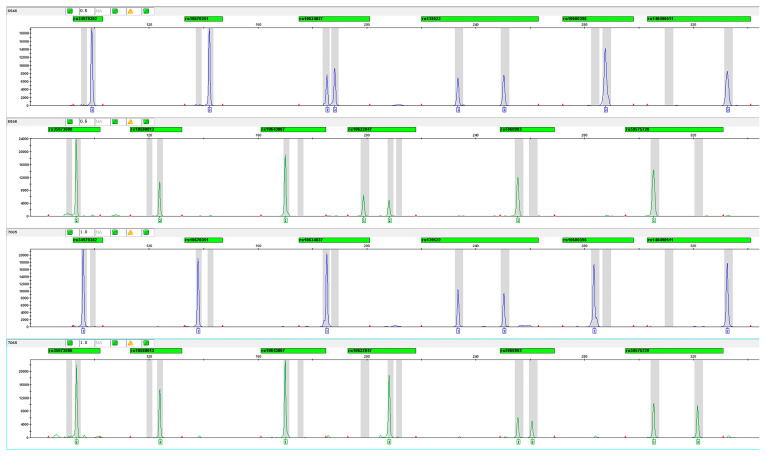
Electropherogram of 12 MCU indel variants using FAM and HEX fluorescence (Sample Nos. 6546 and 7065).

The single primer pairs for capillary electrophoresis (CE) were confirmed to amplify the expected products. After verifying successful amplification for each individual indel variant, all primers were pooled at an equal concentration of 1 μM. Amplifications were performed using 0.25 ng/μL of the 9948 positive control, with primer concentrations optimized based on allelic analysis results from multiplex amplification. Finally, the multiplex primer set was validated using case and control DNA samples. The optimized electropherogram is shown in Figure 2.

### 3.3. Association Between MCU Indel Variants and SCD Susceptibilities

The study included 229 SCD-CAD cases and 598 matched controls from a Southern Han Chinese population. As shown in Table 2, cases had a mean age of 49.52 ± 13.07 years, with males comprising 90.4% of the cohort. No significant differences were observed in age (*p* = 0.18) or sex distribution (*p* = 0.14) between cases and controls. Among cases, physical activity (20.5%) and stress (29.7%) were common triggers of sudden death, while 62.4% exhibited no prior symptoms.

Analysis of 12 candidate indel variants revealed four significant associations with SCD-CAD susceptibility after excluding rs139522 due to its deviation from Hardy–Weinberg equilibrium in controls (*p* = 0.031). As shown in Table 3, protective effects were observed for rs34979382 (OR = 0.57 for del/del genotype) and rs10643067 (OR = 0.21), while rs10670351 showed risk effects (OR = 2.81). Due to the low frequency of the A(ATT)10 allele in rs10622847, we combined it with A(ATT)9 to enhance statistical power. Our analysis demonstrated that both the A(ATT)6 allele (OR = 0.79, 95% CI: 0.63–0.99, *p* = 0.044) and the combined genotype “A(ATT)6/A(ATT)9 + A(ATT)6/A(ATT)10” (OR = 0.54, 95% CI: 0.38–0.75, *p* = 0.0002) were significantly associated with reduced SCD risk compared to A(ATT)9/A(ATT)10 variants. Furthermore, under the recessive model, rs10622847 showed a significant protective effect (OR = 0.60, 95% CI: 0.44–0.82, *p* = 0.001) when comparing combined A(ATT)6-containing genotypes to A(ATT)9/A(ATT)9 + A(ATT)9/A(ATT)10 genotypes.

We performed haplotype analysis of MCU complex indel variants using Haploview v4.2 software. Analysis of variants in *MICU1*, *MCUB*, *MICU2*, and *SMDT1* genes identified 17 distinct haplotypes. Following Bonferroni correction, two risk haplotypes emerged: *MICU1* ‘DEL_rs10670351_-INS_rs10634037_’ (*p* = 0.024) and *MCUB* ‘INS_rs10643067_-INS_rs5860983_’ (*p* = 0.018). In contrast, the *MICU1* ‘INS_rs10670351_-INS_rs10634037_’ haplotype demonstrated a protective effect (*p* = 0.020). Complete haplotype frequencies and association statistics are presented in Table 4.

### 3.4. Bioinformatic Characterization of Four Risk-Associated Indel Variants

**Figure 3:**
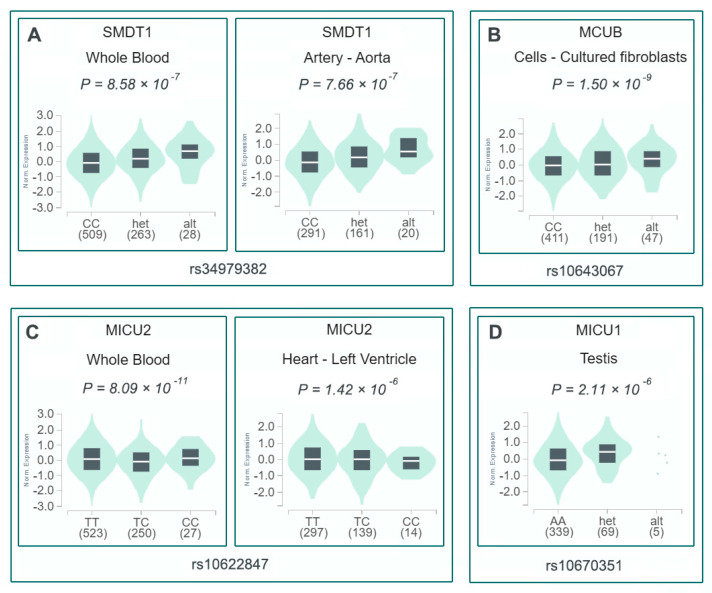
eQTL analysis of MCU complex variants associated with SCD-CAD risk. (**A**) rs34979382 (*SMDT1*) expression in whole blood and aorta. (**B**) rs10643067 (*MCUB*) expression in fibroblasts. (**C**) rs10622847 (*MICU2*) expression in whole blood and left ventricle. (**D**) rs10670351 (*MICU1*) expression in testis.

**Figure 4:**
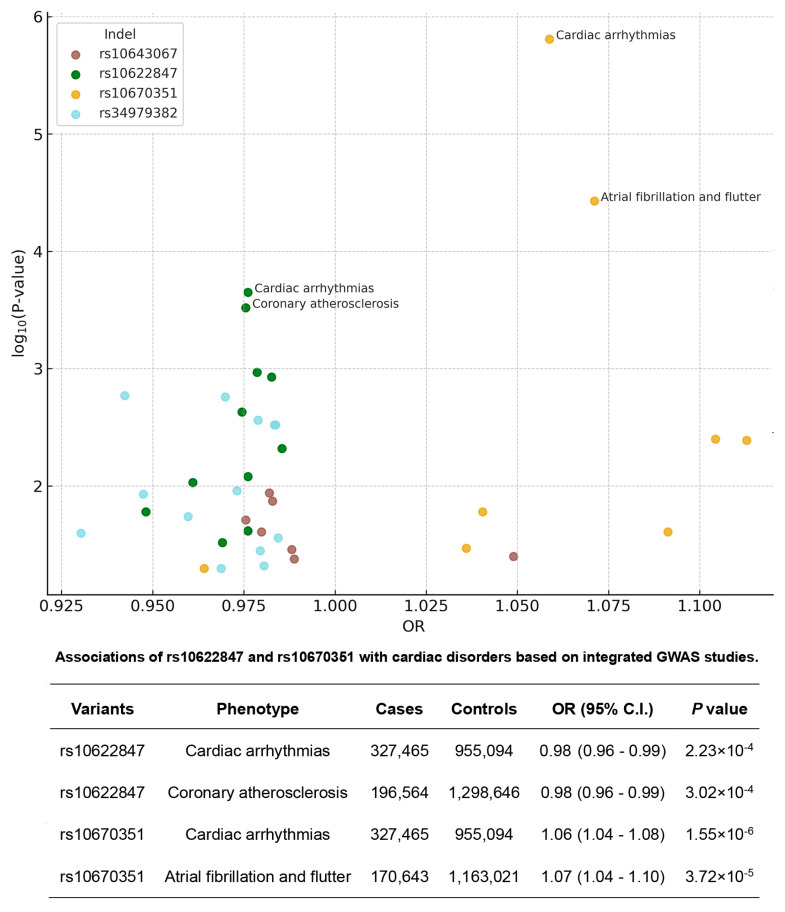
Association of selected MCU complex indels with cardiovascular disease phenotypes. Upper panel: Scatter plots showing significant associations between four MCU complex indel variants and multiple cardiovascular disease outcomes from GWAS databases. Each point represents an individual phenotype association, with size proportional to sample size and color indicating disease category. Lower panel: Detailed correlations for rs10622847 and rs10670351, highlighting their strongest associations with specific cardiac disorders. Data shown include odds ratios, 95% confidence intervals, and *p*-values from meta-analyses of Finngen, UKB, and Million Veteran Program datasets.

eQTL analysis using the GTEx database revealed that all identified variants were significantly associated with expression of their corresponding MCU complex genes (Figure 3). To explore potential cardiovascular disease associations, we analyzed 39 cardiac-related phenotypes from three GWAS databases. The variants demonstrated distinct phenotypic associations: rs34979382 was linked to 13 phenotypes, rs10622847 to 12 phenotypes, and both rs10643067 and rs10670351 to 7 phenotypes each. Scatter plots visualize these cardiac disorder associations, including odds ratios (ORs) and *p*-values. Figure 4 highlights the two indel variants showing the strongest associations with four cardiac disorder phenotypes, along with their detailed statistical information.

### 3.5. Causal Relationship Between SMDT1-Encoded Mitochondrial MCU Regulator Levels and Cardiovascular Diseases

**Figure 5:**
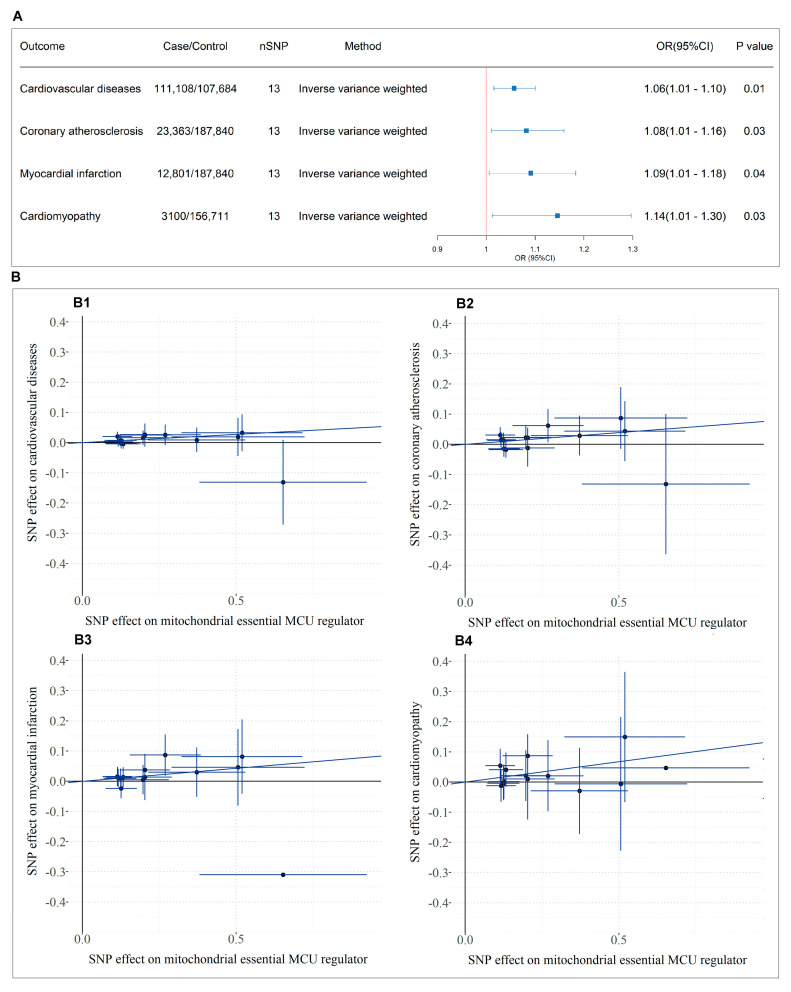
MR analysis of *SMDT1*-encoded mitochondrial MCU regulator levels on cardiovascular outcomes. (**A**) Forest plot of causal effects based on IVW model; (**B**) Scatter plots of variant-outcome associations. B1-B4: Linear regression fits for instrument SNPs with (**B1**) cardiovascular diseases, (**B2**) coronary atherosclerosis, (**B3**) myocardial infarction, and (**B4**) cardiomyopathy.

The MR analysis study design is presented in the bottom panel of Figure 1, where we initially screened and selected 15 eligible SNPs as robust instrumental variables (*F*-statistics > 10) representing SMDT1 levels that satisfied all MR assumptions. Using fixed-effect inverse-variance weighted models as our primary analytical approach, our two-sample MR results demonstrated that genetically predicted *SMDT1*-encoded mitochondrial calcium uniporter regulator levels were significantly associated with increased risks of cardiovascular diseases (OR = 1.06, 95%CI = 1.01–1.10, *p* = 0.008), coronary atherosclerosis (OR = 1.08, 95%CI = 1.01–1.16, *p* = 0.027), myocardial infarction (OR = 1.09, 95%CI = 1.01–1.18, *p* = 0.037), and cardiomyopathy (OR = 1.14, 95%CI = 1.01–1.30, *p* = 0.033), with all findings visually summarized in Figure 5. Comprehensive sensitivity analyses, including MR-Egger, weighted median, and leave-one-out approaches, consistently supported the primary findings, with no evidence of significant horizontal pleiotropy (MR-Egger intercept *p* > 0.05) or undue influence from individual variants.

## 4. Discussion

The MCU complex is the essential pore-forming component that facilitates Ca^2^⁺ entry into the mitochondrial matrix and plays a critical role in maintaining intracellular calcium homeostasis. Comprised of four core subunits, the MCU complex has been increasingly recognized for its pivotal role in cardiovascular regulation. Accumulating evidence suggests that the differential expression of MCU subunits can confer cardiovascular protection, attenuate cardiac cell death, and alleviate vascular inflammation and coronary atherosclerosis [39,40]. However, the potential involvement of genetic polymorphisms in MCU complex genes in the context of cardiovascular disease, particularly SCD-CAD, remains largely unexplored. Our study provides novel insights into the genetic predisposition to SCD-CAD through a comprehensive analysis of MCU complex gene variants in a Southern Han Chinese population. The identification of four significant indel variants (rs34979382, rs10670351, rs10643067, rs10622847) and three risk-associated haplotypes highlights the crucial role of mitochondrial calcium regulation in cardiovascular pathophysiology. These findings are particularly noteworthy as they represent the first reported association between MCU complex polymorphisms and SCD-CAD susceptibility, bridging an important gap in our understanding of genetic risk factors for SCD.

The functional significance of these variants is underscored by their eQTL effects on respective MCU complex genes, as demonstrated in GTEx database analyses. The protective effects observed for rs34979382, rs10622847 and rs10643067, contrasted with the risk conferred by rs10670351, suggest complex regulatory mechanisms governing mitochondrial calcium homeostasis. Specifically, the deletion allele of rs10670351 was associated with reduced expression of MICU1, consistent with previous studies that have highlighted MICU1 upregulation as a key suppressor of endothelial inflammation and atherogenesis [40]. Increased MICU1 expression has also been implicated in protecting against various cardiovascular conditions, including cardiac hypertrophy and hypoxia-induced myocardial injury [41,42]. An equally noteworthy pattern is seen for rs10622847, an intronic indel in *MICU2*. GTEx shows that its protective A(ATT)6/A(ATT)6 genotype up-regulates MICU2 in whole blood yet down-regulates in left-ventricular, forming a bidirectional, tissue-specific eQTL typical of non-coding regulatory variants. This phenomenon is consistent with the well-documented finding that MICU1/MICU2 mRNA ratios vary markedly among tissues—for example, cardiac myocytes versus hepatocytes—thereby shaping the threshold and magnitude of mitochondrial Ca^2^⁺ uptake [43]. Functionally, MICU2 acts as a ‘gain controller’ that elevates the cytosolic Ca^2^⁺ threshold required to open the MCU pore; higher MICU2 expression systemically limits mitochondrial Ca^2^⁺ overload and oxidative stress, whereas a modest reduction can facilitate rapid Ca^2^⁺ entry to match acute energy demand without completely abolishing gatekeeping [44]. This dual role provides a coherent explanation for our observation that the A(ATT)6/A(ATT)6 genotype is cardioprotective: it likely preserves antioxidative capacity in circulating cells while fine-tuning myocardial Ca^2^⁺ handling to avoid lethal overload.

Likewise, for another MCU-regulatory subunit, EMRE, encoded by *SMDT1*, functions as a structural scaffold within the MCU complex and is indispensable for forming a stable Ca^2^⁺ channel. Short-term inhibition of EMRE has been shown to protect the heart from ischemia/reperfusion (I/R) injury [45], aligning with our findings for rs34979382. Interestingly, while some reports have indicated that elevated MCUB expression exerts protective effects in cardiovascular disease [46], our results appear to diverge. MCUB is known as a unique subunit that is typically expressed at low levels under normal physiological conditions but becomes active in response to pathological stimuli [47]. This may explain our observation that the ins allele frequency of rs10643067 was higher in SCD-CAD cases than in controls, implying increased MCUB expression under diseased conditions. Haplotype-block analysis has been shown to improve detection power by 15–50% compared to single-locus analyses [48]. Our haplotype analysis confirmed this advantage, revealing that combinations of variants may exert synergistic effects on disease susceptibility. This was particularly evident in the *MICU1* locus, where the ‘DEL-INS’ haplotype was associated with elevated risk, while the ‘INS-INS’ haplotype demonstrated a protective role.

The biological plausibility of our findings is supported by extensive evidence linking mitochondrial calcium homeostasis to key cardiac processes. The heart is heavily reliant on mitochondrial ATP production to meet its high energy demands, and mitochondrial Ca^2^⁺ influx plays a central role in excitation–metabolism coupling. However, excessive mitochondrial calcium accumulation may induce mitochondrial dysfunction, increase reactive oxygen species (ROS) production, and trigger cell death pathways. Acute mitochondrial Ca^2^⁺ overload is believed to alter mitochondrial membrane permeability and has been implicated in myocardial infarction and I/R injury [13,39]. Furthermore, disturbances in Ca^2^⁺ homeostasis are recognized as a hallmark of heart failure, contributing to compromised contractility and progressive cardiac dysfunction [12]. Emerging evidence also suggests a role for mitochondrial Ca^2^⁺ regulation in atherosclerosis progression. Under physiological conditions, mitochondrial Ca^2^⁺ signaling interfaces with lipid metabolism and inflammatory cascades—particularly via the MAPK signaling pathway [49]. In hepatic cells, MCU deficiency has been associated with increased lipid accumulation [50], and a murine atherosclerosis model demonstrated that MCU knockdown mitigated endothelial injury, further implicating the MCU complex in atherogenesis [51]. As for animal models, *MICU2*^−/−^ mutant mice displayed left atrial enlargement, and the mutated cardiomyocytes exhibited delayed sarcomere relaxation and slower cytosolic calcium reuptake kinetics, suggesting diastolic dysfunction [52]. These models highlight the essential role of the MCU complex in protecting cardiomyocytes from cell death and maintaining cardiac function, particularly under pathological conditions such as ischemia/reperfusion injury. As the MCU complex governs mitochondrial Ca^2^⁺ influx, its central role in regulating [Ca^2^⁺]ₘ, ATP production, and ROS generation provides multiple potential pathways through which the identified variants could influence SCD risk.

In this context, our MR findings, suggesting a causal relationship between *SMDT1*-encoded MCU levels and cardiovascular diseases, align with previous literature on MCU function, while significantly extending this understanding into the realm of genetic predisposition to SCD-CAD. This represents a significant methodological advancement, establishing for the first time a causal relationship between genetically predicted *SMDT1*-encoded MCU regulator levels and various cardiovascular diseases. The consistent associations across multiple cardiac phenotypes, supported by robust sensitivity analyses, strongly suggest that mitochondrial calcium dysregulation is not merely correlated with but may actively contribute to cardiovascular pathogenesis. This finding has useful implications for therapeutic targeting, as it suggests that modulation of MCU complex activity could potentially influence disease progression.

Several limitations should be acknowledged. The relatively small sample size of SCD-CAD cases, while understandable given the challenges of sample collection in a forensic context, may limit the generalizability of our findings. The European ancestry of MR reference populations raises questions about the trans-ethnic applicability of these results. Additionally, the absence of functional validation studies means the precise molecular mechanisms underlying these associations remain to be elucidated. Despite these limitations, our study makes valuable contributions to the field. The development of a novel capillary electrophoresis-based genotyping system for MCU complex variants provides a valuable tool for future research and potential clinical applications. The identification of specific risk and protective haplotypes offers new possibilities for personalized risk assessment. Most importantly, our findings establish mitochondrial calcium regulation as a key pathway in SCD-CAD pathogenesis, opening new avenues for therapeutic intervention and prevention strategies.

Future research directions should include: (1) replication studies in larger, multi-ethnic cohorts; (2) functional characterization of the identified variants; (3) investigation of gene-environment interactions; and (4) exploration of potential therapeutic interventions targeting the MCU complex. Such studies will be crucial for translating these genetic findings into clinical applications that could ultimately reduce the burden of SCD.

## 5. Conclusions

This study revealed that specific indel variants in MCU complex genes significantly modulate SCD-CAD risk in the Southern Han Chinese population. These variants function as eQTLs for their respective genes, with haplotype analysis revealing synergistic risk/protective effects. MR analysis demonstrated a causal relationship between elevated *SMDT1*-encoded MCU regulator levels and increased cardiovascular disease risk. Our findings implicated mitochondrial calcium dysregulation as a key mechanistic pathway in SCD-CAD and highlight the MCU complex as a promising therapeutic target. Further validation in diverse cohorts and functional studies are warranted to translate these genetic insights into clinical applications.

## Figures and Tables

**Table 1:** The basic information of candidate indels selected from the MCU complex.

Gene Symbol	Indel ID	Chr: Position (GRCh38)	MAF (RGC—Million Exome Variant Browser)	Alleles
*SMDT1*	rs34979382	22:42218818–42218821	0.35	dupGTT
*MICU1*	rs10670351	10:72985345–72985347	0.25	insTGTT
*MICU1*	rs10634037	10:73062986–73062989	0.33	dupCCC
*SMDT1*	rs139522	22:41266253–41266255	0.20	insCCTGAAGCAACCACAGC
*MICU3*	rs10680396	8:17120219–17120220	0.21	insGATT
*SMDT1*	rs140490511	22:42068821–42068844	0.14	dupGGAATTAGC(A)_4_CTAACACCT
*MICU2*	rs35073080	13:21422625–21422636	0.22	delATT
*MICU2*	rs10580013	13:21371494–21371499	0.23	delAATA
*MCUB*	rs10643067	4:109572694–109572697	0.10	insGACTT
*MICU2*	rs10622847	13:21600794–21600812	0.39	dup(ATT)_3_/dup(ATT)_4_
*MCUB*	rs5860983	4:109630153–109630157	0.29	del(T)_5_
*SMDT1*	rs59575728	22:42042421–42042442	0.43	delACTTGAGTCA(TCT)_2_

Chr, chromosome; MAF, Minor allele frequency.

**Table 2:** Demographic characteristics of SCD-CAD cases and controls recruited from the Southern Han Chinese population.

Characteristic	SCD-CAD	SCD Matched Controls	*p*-Value
No. of individuals	229	598	
Sex, No.			
Male	207	518	0.14 ^a^
Female	22	80	
Age, mean ± SD (range)			
Overall	49.52 ± 13.07 (23–86)	48.09 ± 14.04 (16–90)	0.18 ^b^
Males	48.66 ± 12.48 (23–86)	47.16 ± 13.28 (16–90)	0.16 ^b^
Females	57.55 ± 15.88 (27–85)	54.11 ± 17.20 (24–89)	0.40 ^b^
Events at sudden death (SD)			
Nonspecific	96		
Physical activity	47		
Stress	68		
Sleep	18		
Symptoms before SD			
None	143		
Others	86		
Megalothymus			
Positive	5		
Negative	224		

^a^ Chi square test for differences between SCD-CAD and controls. ^b^ Two-sided two-sample *t* test between SCD-CAD and controls.

**Table 3:** Associations between 11 indel variants and SCD-CAD susceptibility in case control sets recruited during 2012–2020 *.

Indel	Genetic Model	Genotype	Cases	(%)	Control	(%)	OR (95% C.I.) ^a^	*p*-Value
rs34979382	Codominant model	ins/ins	34	15.35	58	9.70	1.00 (Reference)	
		ins/del	91	39.91	234	39.13	0.66 (0.41–1.08)	0.098
		del/del	102	44.74	306	51.17	0.57 (0.35–0.92)	**0.020**
		*P*_trend_						**0.029**
	Recessive model	ins/ins	34	14.98	58	9.70	1.00 (Reference)	
		del/del + ins/del	193	85.02	540	90.30	0.61 (0.39–0.96)	**0.031**
	Additive model	ins allele	159	35.02	350	29.26	1.00 (Reference)	
		del allele	295	64.98	846	70.74	0.77 (0.61–0.97)	**0.024**
rs10670351	Codominant model	ins/ins	8	3.51	54	9.03	1.00 (Reference)	
		ins/del	95	41.67	244	40.80	2.63 (1.21–5.73)	**0.012**
		del/del	125	54.82	300	50.17	2.81 (1.30–6.08)	**0.006**
		*P*_trend_						**0.038**
	Recessive model	ins/ins	8	3.51	54	9.03	1.00 (Reference)	
		del/del + ins/del	220	96.49	544	90.97	2.73 (1.28–5.83)	**0.007**
	Additive model	ins allele	111	24.34	352	29.43	1.00 (Reference)	
		del allele	345	75.66	844	70.57	1.30 (1.01–1.66)	**0.040**
rs10634037	Codominant model	ins/ins	19	8.52	55	9.20	1.00 (Reference)	
		ins/del	91	40.81	254	42.47	1.04 (0.58–1.84)	0.901
		del/del	113	50.67	289	48.33	1.13 (0.64–1.99)	0.667
		*P*_trend_						0.552
	Recessive model	ins/ins	19	8.52	55	9.20	1.00 (Reference)	
		del/del + ins/del	204	91.48	543	90.80	1.09 (0.63–1.88)	0.763
	Additive model	ins allele	129	28.92	364	30.43	1.00 (Reference)	
		del allele	317	71.08	832	69.57	1.08 (0.85–1.37)	0.552
rs10680396	Codominant model	ins/ins	14	6.42	30	5.03	1.00 (Reference)	
		ins/del	73	33.49	210	35.24	0.75 (0.37–1.48)	0.400
		del/del	131	60.09	356	59.73	0.79 (0.41–1.53)	0.483
		*P*_trend_						0.828
	Recessive model	ins/ins	14	6.42	30	5.03	1.00 (Reference)	
		del/del + ins/del	204	93.58	566	94.97	0.77 (0.40–1.49)	0.438
	Additive model	ins allele	101	23.17	270	22.65	1.00 (Reference)	
		del allele	335	76.83	922	77.35	0.97 (0.75–1.26)	0.827
rs140490511	Codominant model	ins/ins	163	74.43	425	71.19	1.00 (Reference)	
		ins/del	49	22.37	156	26.13	0.82 (0.57–1.18)	0.287
		del/del	7	3.20	16	2.68	1.14 (0.46–2.82)	0.776
		*P*_trend_						0.507
	Recessive model	ins/ins	163	74.43	425	71.19	1.00 (Reference)	
		del/del + ins/del	56	25.57	172	28.81	0.85 (0.60–1.21)	0.361
	Additive model	ins allele	375	85.62	1006	84.25	1.00 (Reference)	
		del allele	63	14.38	188	15.75	0.90 (0.66–1.22)	0.499
rs35073080	Codominant model	ins/ins	104	47.71	279	47.45	1.00 (Reference)	
		ins/del	87	39.91	246	41.84	0.95 (0.68–1.32)	0.756
		del/del	27	12.38	63	10.71	1.15 (0.70–1.90)	0.587
		*P*_trend_						0.791
	Recessive model	ins/ins	104	47.71	279	47.45	1.00 (Reference)	
		del/del + ins/del	114	52.29	309	52.55	0.99 (0.73–1.35)	0.948
	Additive model	ins allele	295	67.66	804	68.37	1.00 (Reference)	
		del allele	141	32.34	372	31.63	1.03 (0.82–1.31)	0.787
rs10580013	Codominant model	ins/ins	109	48.66	281	47.23	1.00 (Reference)	
		ins/del	89	39.73	252	42.35	0.91 (0.66–1.26)	0.575
		del/del	26	11.61	62	10.42	1.08 (0.65–1.80)	0.764
		*P*_trend_						0.962
	Recessive model	ins/ins	109	48.66	281	47.23	1.00 (Reference)	
		del/del + ins/del	115	51.34	314	52.77	0.94 (0.69–1.28)	0.714
	Additive model	ins allele	307	68.53	814	68.40	1.00 (Reference)	
		del allele	141	31.47	376	31.60	0.99 (0.79–1.26)	0.962
rs10643067	Codominant model	ins/ins	5	2.21	3	0.50	1.00 (Reference)	
		ins/del	45	19.91	91	15.24	0.30 (0.07–1.30)	0.089
		del/del	176	77.88	503	84.26	0.21 (0.05–0.89)	**0.020**
		*P*_trend_						**0.012**
	Recessive model	ins/ins	5	2.21	3	0.50	1.00 (Reference)	
		del/del + ins/del	221	97.79	594	99.50	0.22 (0.05–0.94)	**0.026**
	Additive model	ins allele	55	12.17	97	8.12		
		del allele	395	87.83	1097	91.88	0.64 (0.45–0.90)	**0.011**
rs5860983	Codominant model	ins/ins	20	9.01	39	6.54	1.00 (Reference)	
		ins/del	71	31.98	237	39.77	0.58 (0.32–1.07)	0.077
		del/del	131	59.01	320	53.69	0.80 (0.45–1.42)	0.443
		*P*_trend_						0.563
	Recessive model	ins/ins	20	9.01	39	6.54	1.00 (Reference)	
		del/del + ins/del	202	90.99	557	93.46	0.71 (0.40–1.24)	0.226
	Additive model	ins allele	111	25.00	315	26.43	1.00 (Reference)	
		del allele	333	75.00	877	73.57	1.08 (0.84–1.38)	0.559
rs59575728	Codominant model	ins/ins	48	21.92	146	24.46	1.00 (Reference)	
		ins/del	102	46.58	287	48.07	1.08 (0.73–1.61)	0.700
		del/del	69	31.50	164	27.47	1.28 (0.83–1.97)	0.261
		*P*_trend_						0.249
	Recessive model	ins/ins	48	21.92	146	24.46	1.00 (Reference)	
		del/del + ins/del	171	78.08	451	75.54	1.15 (0.80–1.67)	0.450
	Additive model	ins allele	198	45.21	579	48.49	1.00 (Reference)	
		del allele	240	54.79	615	51.51	1.14 (0.92–1.42)	0.239
rs10622847	Codominant model	A(ATT)_9_/A(ATT)_9_ + A(ATT)_9_/A(ATT)_10_	107	47.56	209	35.13	1.00 (Reference)	
		A(ATT)_6_/A(ATT)_9_ + A(ATT)_6_/A(ATT)_10_	82	36.44	299	50.25	0.54 (0.38–0.75)	**2 × 10^−4^**
		A(ATT)_6_/A(ATT)_6_	36	16.00	87	14.62	0.81 (0.51–1.27)	0.356
		*P*_trend_						**0.042**
	Recessive model	A(ATT)_9_/A(ATT)_9_ + A(ATT)_9_/A(ATT)_10_	107	47.56	209	35.13	1.00 (Reference)	
		A(ATT)_6_/A(ATT)_9_ + A(ATT)_6_/A(ATT)_10_ + A(ATT)_6_/A(ATT)_6_	118	52.44	386	64.87	0.60 (0.44–0.82)	**0.001**
	Additive model	A(ATT)_9_ + A(ATT)_10_	296	65.78	717	60.25	1.00 (Reference)	
		A(ATT)_6_	154	34.22	471	39.75	0.79 (0.63–0.99)	**0.044**

* rs139522 was omitted during analysis due to its disagreement with the Hardy–Weinberg equilibrium (*p* = 0.031). ^a^ Adjusted by age and gender factors. Bold text indicates statistical significance (*p* < 0.05). CI, confidence interval; OR, odds ratio.

**Table 4:** Result of MCU complex haplotype analysis in SCD-CAD cases and controls.

Indel Variants Haplotype					Freq (Case)	Freq (Control)	χ^2^	*p*-Value
*MICU1*:rs10670351|rs10634037								
	DEL		DEL		62.63	61.31	0.244	0.6211
	INS		INS		16.07	21.18	5.424	**0.020**
	DEL		INS		13.03	9.26	5.072	**0.024**
	INS		DEL		8.27	8.25	0.001	0.992
*MCUB*:rs10643067|rs5860983								
	DEL		DEL		64.40	66.55	0.670	0.413
	DEL		INS		23.43	25.33	0.636	0.425
	INS		DEL		10.58	7.03	5.642	**0.018**
	INS		INS		1.59	1.10	0.634	0.426
*MICU2*:rs10580013|rs35073080|rs10622847								
	INS	INS		INS	58.25	55.47	1.022	0.312
	DEL	DEL		DEL	24.25	26.66	0.991	0.320
	INS	INS		DEL	9.65	12.67	2.854	0.091
	DEL	DEL		INS	6.26	4.59	1.926	0.165
*SMDT1*:rs59575728|rs140490511|rs34979382								
	INS	INS		DEL	29.24	31.57	0.826	0.363
	DEL	INS		INS	32.91	27.88	3.991	0.046
	DEL	INS		DEL	21.88	23.44	0.452	0.502
	INS	DEL		DEL	14.28	15.50	0.382	0.537
	INS	INS		INS	1.64	1.39	0.141	0.707

*p*-values have maintained their significance after Bonferroni correction (0.05/2 = 0.025 for *MICU1* and *MCUB*; 0.05/3 = 0.0167 for *MICU2* and *SMDT1*). χ^2^ chi-square value, *p* ≤ Bonferroni correction result and bold text considered as statistically significant.

## Data Availability

The summary-level data are available from the IEU GWAS database (https://gwas.mrcieu.ac.uk/, accessed on 11 October 2024). The data that support the findings of this study are also available from the corresponding author upon reasonable request.

## Supplementary Materials

The following supporting information can be downloaded at: https://www.mdpi.com/article/10.3390/cells14100728/s1, Table S1: The specific methods for DNA extraction, PCR reactions and genotyping; Table S2: Publicly available MR Base GWAS catalog used for bidirectional Two-sample Mendelian randomization study. Table S3: Specific information of candidate Indel variants based on five databases.

## Abbreviations

The following abbreviations are used in this manuscript:

SCD	sudden cardiac death
SCD-CAD	coronary artery disease-related sudden cardiac death
MCU	mitochondrial calcium uniporter
IMM	the inner mitochondrial membrane
MCUB	the dominant-negative β-subunit
MICU	the mitochondrial calcium uptake
EMRE	the essential MCU regulator
MCUR1	the MCU regulator 1
ROS	reactive oxygen species
I/R	ischemia-reperfusion
CE	capillary electrophoresis
STR	short tandem repeat
Indel	insertions and deletion
SNP	single nucleotide polymorphisms
MR	Mendelian randomization
GWAS	genome-wide association studies
GTEx	the genotype-tissue expression
SMDT1	single-pass membrane protein with aspartate rich tail 1
eQTL	expression quantitative trait loci
MAF	minor allele frequency
PAGE	polyacrylamide gel electrophoresis
LD	Linkage disequilibrium
RFU	relative fluorescence units
HWE	Hardy–Weinberg equilibrium
OR	odds ratio
CI	confidence interval
IVW	inverse-variance weighted
MR-PRESSO	Mendelian randomization pleiotropy residual sum and outlier
